# Scene-Level Geographic Image Classification Based on a Covariance Descriptor Using Supervised Collaborative Kernel Coding

**DOI:** 10.3390/s16030392

**Published:** 2016-03-18

**Authors:** Chunwei Yang, Huaping Liu, Shicheng Wang, Shouyi Liao

**Affiliations:** 1 High-Tech Institute of Xi’an, Xi’an 710025, China; wangsching@163.com (S.W.); liaoshouyi123@163.com (S.L.); 2 Department of Computer Science and Technology, Tsinghua University, Beijing 100084, China; hpliu@tsinghua.edu.cn

**Keywords:** scene-level geographic image classification, covariance descriptor, collaborative kernel coding

## Abstract

Scene-level geographic image classification has been a very challenging problem and has become a research focus in recent years. This paper develops a supervised collaborative kernel coding method based on a covariance descriptor (covd) for scene-level geographic image classification. First, covd is introduced in the feature extraction process and, then, is transformed to a Euclidean feature by a supervised collaborative kernel coding model. Furthermore, we develop an iterative optimization framework to solve this model. Comprehensive evaluations on public high-resolution aerial image dataset and comparisons with state-of-the-art methods show the superiority and effectiveness of our approach.

## 1. Introduction

**Figure 1:**
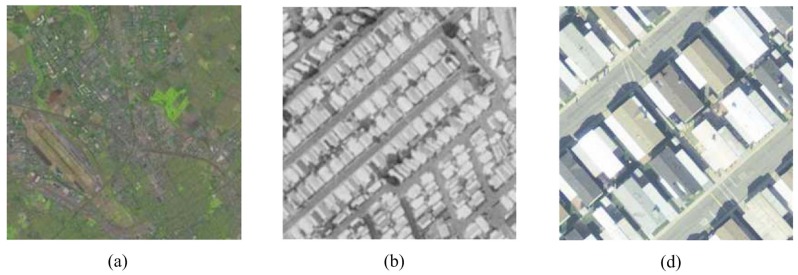
Images with a resolution of: (**a**) 30 m; (**b**) 1 m; (**c**) 0.3 m.

Nowadays, high spatial resolution remote sensing images are easily acquired thanks to the rapid development of satellite and remote sensing technology, which has endowed us with the opportunity to interpret, analyze and understand the image. As a fundamental research area of remote sensing image analysis, scene-level geographic image classification is of great importance for land use and land cover (LULC) image classification [1,2,3], semantic interpretations of images [4], geographic image retrieval [5,6,7] and forest type mapping [8], which has drawn increasing attention and scholars’ study [1,2,3,5,9,10,11,12,13]. Figure 1 shows geographic images whose spatial resolution is 30 m, 1 m and 0.3 m, respectively.

However, finding an efficient representation of the scene-level image is a challenging problem. The bag of visual words (BOVW) model [14] is one of the most successful models. The works in [2,5] detailed the application of BOVW on the scene-level image classification task. As is illustrated in [2,5], BOVW can represent the image by compact representation through a visual word counts histogram and provides further invariance to the image transformations. However, the tradeoff between invariance and discriminability is controlled by the visual dictionary size. What is more, BOVW disregards the information about the spatial layout of the features, which is of great importance to scene-level image classification [2,15,16]. In order to overcome this shortcoming, one successful extension of BOVW is spatial pyramid matching (SPM) [16], which partitions the image into increasing finer sub-images and computes histograms of local features from each sub-image. Although SPM is a computationally-efficient extension of BOVW and shows superior performance, it does not consider the relative spatial arrangement and only characterizes the absolute location of the visual words in an image. From this point of view, SPM also limits the descriptive ability of the scene-level geographic image representation. Hence, two new image representation models, which are termed spatial co-occurrence kernel (SCK) [1] and spatial pyramid co-occurrence kernel (SPCK) [2], are proposed by Yang and Newsam. What is more, in order to capture the absolute and relative spatial relationships of BOVW, a pyramid of spatial relations (PSR) model is developed by Chen and Tian. The work in [17] points out that the computational complexities of SCK and SPCK are high because of the need to use nonlinear Mercer kernels and developed a linear form of the SCK. Besides, [10] proposed an unsupervised feature learning method, in which the new sparse representations of the feature descriptors are generated by the low-level feature descriptors.

**Figure 2:**
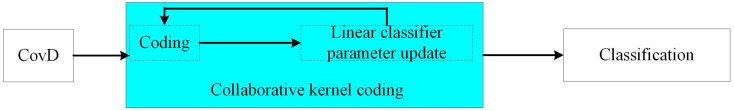
Illustration of the supervised collaborative kernel coding model.

On the other hand, the covariance descriptor (covd) proposed by by Tuzel [18] can be used for feature representation of the image, which has been extensively adopted in vast computer vision tasks, e.g., texture discrimination [18], visual saliency estimation [19], object detection [18,20] and object tracking [21]. Covd is a covariance matrix of different features, e.g., color, gradient and spatial location, and it holds certain rotation and scale invariance. However, how to model and compute covd still remains a key problem. We all know that covd lies in the Riemannian manifold, which is a non-Euclidean space. As a result, traditional mathematical modeling and computation in Euclidean space cannot be directly utilized, which results in a great challenge. In [22], a discriminative learning method is developed to formulate the classification problem on Riemannian space by covd, which presents a kernel function and a log-Euclidean distance metric to solve Riemannian-Euclidean transformation. In [23], a coding strategy is introduced, and the descriptor can be transformed into a new feature; and then, extreme learning machine (ELM) can be used for dynamic texture video classification. However, such a method separately optimizes the reconstruction error of the coding and the classification error of ELM, and the design stage of coding and the classifier are totally independent. In order to solve this problem, a supervised collaborative kernel coding approach incorporating the linear classifier supervised term that can optimize both the reconstruction error and the linear classifier simultaneously is developed. There are three contributions as follows:

1. A supervised collaborative kernel coding model, illustrated in Figure 2, is proposed. This model can not only transform the covd to a discriminative feature representation, but also can obtain the corresponding linear classifier.
2. An iterative optimization framework is introduced to solve the supervised collaborative kernel coding model.
3. Experiments on public high-resolution aerial image dataset validate that the proposed supervised collaborative kernel coding model derives a satisfying performance on the scene-level geographic image classification.

The paper is organized as follows: After a review of our proposed methodology in Section 2, Section 3 shows the iterative optimization approach. In Section 4 and Section 5, we give the experiments and conclusions.

## 2. Overview of the Methodology

**Figure 3:**
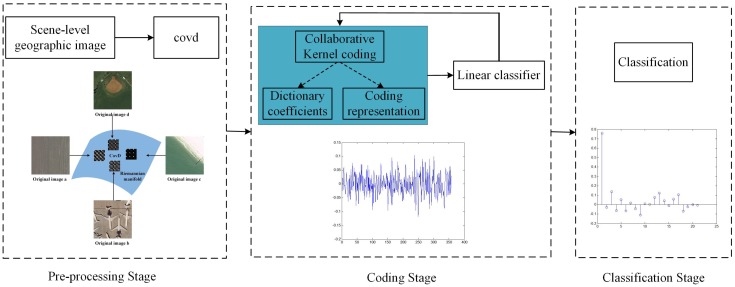
The overview of the proposed method. covd, covariance descriptor.

Figure 3 shows the overview of the proposed method, which consists of 3 stages, the pre-processing stage, coding stage and classification stage. In the pre-processing stage, covd is extracted as the initial feature representation of the scene-level geographic image. Then, in the coding stage, the supervised collaborative kernel coding strategy involving dictionary coefficients, the coding representation phase and the linear classification phase is presented. Finally, in the classification stage, based on the dictionary coefficients and learned linear classifier, a label vector can be simply derived through the linear classifier, the index corresponding to the largest value of which is the label of a testing scene-level geographic image.

### 2.1. Covariance Descriptor

Covd was first proposed by Tuzel *et al.* [18] as a compact descriptor. Formally, let ${\left\{f_{k}\right\}}_{k=1,\cdots ,d}$ be a feature vector denoting the feature points of *p*-dimension as color, gradient filter response, *etc*. Then, a covd C of s×s dimensions of an image can be described as:

(1) $$C=\frac{1}{d-1}\sum_{k=1}^{d}\left(f_{k}-v\right){\left(f_{k}-v\right)}^{T}$$

where *d* and v denote the pixel number and the mean value, respectively.

The feature vector f is established using the image intensity of each channel, the norm of the first and second derivatives of intensity in the *x* and *y* directions. As for a geographic image, a feature vector $f_{x,y}={\left[c_{R,x,y}^{T},c_{G,x,y}^{T},c_{B,x,y}^{T}\right]}^{T}$ of 15 dimensions is computed at each pixel (x,y), and here, $c_{C,x,y}=[I_{C,x,y},|\frac{\partial I_{C}}{\partial x}\left|,\right|\frac{\partial^{2}I_{C}}{\partial^{2}x}\left|,\right|\frac{\partial I_{C}}{\partial y}\left|,\right|\frac{\partial^{2}I_{C}}{\partial^{2}y}\left|\right]$, where $I_{C}$ and $C\in \left\{R,G,B\right\}$ denote the the *C* channel intensity image and the channel of the color, respectively.

The work in [18] points out that covd has at least three characteristics: (1) it is enough to describe the image of different poses and views; (2) multiple features can be fused in a natural way through covd, the diagonal and non-diagonal elements of which describe the variance and correlations of different features, respectively; (3) comparing to other descriptors, such as raw values and the histogram, covd is low-dimensional, and it has only $\frac{s^{2}+s}{2}$ different values due to symmetry.

**Figure 4:**
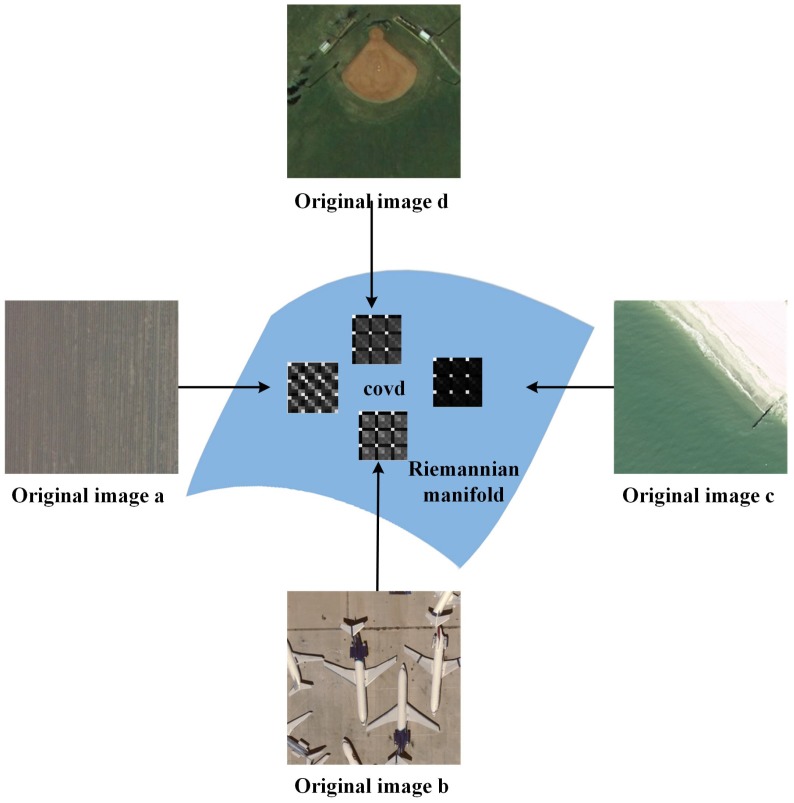
Sample geographic images and corresponding covariance descriptor (covd) features.

Nevertheless, covd is a symmetric positive definite matrix. The key issue for a symmetric positive definite matrix is how to model and compute it. As is illustrated in Figure 4, covd lies in a Riemannian manifold [24], which is not a Euclidean space.

Accordingly, the mathematical modeling of covd is not the same as what we usually do in the Euclidean space. Here, we adopt the idea of Ruiping Wang [22] and compute the distance of two covds $C_{1}$ and $C_{2}$ using log-Euclidean distance [25,26]:

(2) $$d\left(C_{1},C_{2}\right)=||\mathrm{logm}\left(C_{1}\right)-\mathrm{logm}\left(C_{2}\right){\left|\right|}_{F}$$

where logm is the logarithm computation of the matrix and $\left|\right|\cdot {\left|\right|}_{F}$ denotes the Frobenius norm.

Moreover, there is a tricky problem regarding how to use covd in the geographic image classification. It is a fact that covd lies in a non-Euclidean space; thus, the traditional linear classifier based on Euclidean space cannot be directly utilized. Therefore, in the following, how to solve this problem is the theme.

### 2.2. Supervised Collaborative Kernel Coding Model

**Figure 5:**
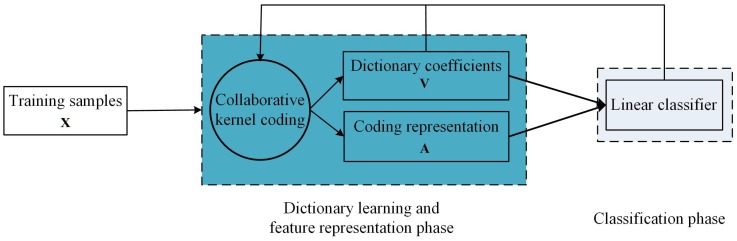
The illustration of the proposed model.

As is shown in Figure 5, here, we propose a supervised collaborative kernel coding model, which consists of two jointly working components: (1) the dictionary learning and feature representation phase; and (2) the linear classification phase. First, the linear classifier is incorporated into the dictionary learning and feature representation phase, making the resulting coding vector A more discriminative. Then, based on the coding vector A, the linear classifier W is derived. In this way, the objectives function in each phase are combined into a unified optimization framework, through which a collaborative coding vector and the corresponding linear classifier can be simultaneously obtained. At last, based on the dictionary coefficients V, testing signal $s_{i}$ is transformed into a feature vector, which is used for linear classification directly.

Denote ${\left\{x_{i}\right\}}_{i=1}^{N}\in H$ as the training samples, where H is a Riemannian manifold. Through the proper mapping function, ${\left\{x_{i}\right\}}_{i=1}^{N}$ are mapped into a higher dimensional space. Namely, let Φ(·):H→P be the nonlinear mapping process from the original space H into a high or infinite dimensional space P. For convenience, the dimension of P is denoted as $\tilde{m}$. The mapping function here is associated with a kernel $\kappa \left(x_{i},x_{j}\right)=<\Phi^{T}\left(x_{i}\right),\Phi \left(x_{j}\right)>$, where $x_{i},x_{j}\in H$. As for covd computation, the Gaussian kernel is chosen as the mapping function for its superior performance in vast computer vision tasks [27]:

(3) $$\kappa \left(x_{i},x_{j}\right)=\exp(-\beta ||\mathrm{logm}\left(x_{i}\right)-\mathrm{logm}\left(x_{j}\right){\left|\right|}^{2})\;$$

where the decay parameter *β* is empirically set as 0.02 and $\kappa (x_{i},x_{j})$ is the Gaussian kernel between two samples $x_{i}$ and $x_{j}$.

The aim of dictionary learning is to empirically learn a dictionary adapted to the training sample set; therefore, we need to determine some atoms $d_{1},\cdots ,d_{K}\in P$ to represent the training samples, where *K* is the dictionary size and K<N. Let $\Phi (X)=\left[\Phi {\left(x\right)}_{1},\cdots ,\Phi {\left(x\right)}_{N}\right]\in R^{\tilde{m}\times K}$, and the kernel dictionary learning process can be formulated as:

(4) $$\min_{D,A}||\Phi (X)-{\Phi (D)A||}_{2}^{2}+{\lambda ||A||}_{2}^{2}\;$$

where $A\in R^{K\times N}$ is the coding matrix and *λ* is the penalty parameter.

Thanks to the kernel trick [28,29], through the mapping function Φ(·), the problem on the Riemannian manifold can be transformed to a collaborative coding problem in the Euclidean space. Nevertheless, since the number of dictionary atoms $d_{j}$ may be infinite, there exists a new challenge to the dictionary learning process in such a formulation. Fortunately, [30,31] prove that the dictionary D can be represented as D=Φ(X)V, where $V\in R^{N\times K}$ is a coefficient matrix. This indicates that the training samples can linearly represent the dictionary in the feature space. As a result, Equation (4) can be reformulated as:

(5) $$\min_{V,A}||\Phi (X)-{\Phi (X)VA||}_{2}^{2}+{\lambda ||A||}_{2}^{2}\;$$

Such a formulation provides two significant advantages: (1) the dictionary learning process becomes searching the matrix V; (2) for any kernel function, this formulation reduces the dictionary learning process to linear problems.

Now, we propose a novel objective function combining both the collaborative kernel coding phase and classification phase as:

(6) $$\min_{V,A,W}||\Phi (X)-{\Phi (X)VA||}_{2}^{2}+{\lambda ||A||}_{2}^{2}+\eta ||L-{\mathrm{WA}||}_{2}^{2}+{\rho ||W||}_{2}^{2}\;$$

where $||\Phi (X)-{\Phi (X)VA||}_{2}^{2}$ and $||L-{\mathrm{WA}||}_{2}^{2}$ denotes the reconstruction error and the linear classification error, respectively, and W represents the classifier parameters. *η*, *λ* and *ρ* are all penalty parameters.

The derived dictionary through this formulation can generate more discriminative codes A, which is of great importance to the performance of the classifier and also adaptive to the underlying structure of training samples. The resulting codes A are then directly used for classification.

For a testing sample $s_{i}$, through Equation (7), the feature representation code $z_{i}$ is firstly computed with dictionary coefficients V. Then, in order to derive the label vector, we can use $l_{i}={\mathrm{Wz}}_{i}$. The index corresponding to the largest value of $l_{i}$ is the label of $s_{i}$.

(7) $$\min_{z_{i}}||\Phi (s)-\Phi (X)Vz_{i}{\left|\right|}_{2}^{2}+\lambda ||z_{i}{\left|\right|}_{2}^{2}\;$$

## 3. Optimization Algorithm

There are three variables as V, A and W in the objective function Equation (6). Here, an iterative optimization algorithm for each variable by fixing the other two is introduced. (Equation (6) is denoted as F(V,A,W), and the obtained variables from the *k*-th and (k+1)-th iteration are denoted as the subscripts (k) and (k+1), respectively, and k=0,⋯,N-1).

Step 1: Initialization. We randomly set coefficient matrix $V_{0}\in R^{N\times K}$. Next, we compute the corresponding coding coefficient A by taking the derivative of A of Equation (6):

(8) $$A_{0}={\left(V^{T}K\left(X,X\right)V+\lambda I\right)}^{-1}V^{T}K^{T}\left(X,X\right))$$

where K(X,X) is an N×N square matrix of which the (i,j)-th element is $\kappa (x_{i},x_{j})$.

Step 2: Fixing A, taking the derivative of V:

(9) $$\frac{\partial F(V,A_{\left(k\right)},W_{\left(k\right)})}{\partial V}=0$$

Additionally, the corresponding solution is:

(10) $$V_{\left(k+1\right)}=A_{\left(k\right)}^{T}{\left(A_{\left(k\right)}A_{\left(k\right)}^{T}\right)}^{-1}$$

Step 3: Fixing V and A, taking the derivative of W, we can derive the optimal solution of W.

(11) $$\frac{\partial F(V_{\left(k+1\right)},A_{\left(k+1\right)},W)}{\partial W}=0\;$$

(12) $$W_{\left(k+1\right)}={\left(\eta A_{\left(k+1\right)}A_{\left(k+1\right)}^{T}+\rho I\right)}^{-1}\eta IA_{\left(k+1\right)}^{T}\;$$

Step 4: Fixing V and W, and taking the derivative of A:

(13) $$\frac{\partial F(V_{\left(k+1\right)},A,W_{\left(k\right)})}{\partial A}=0\;$$

Then, the optimal solution of A is:

(14) $$A_{\left(k+1\right)}={\left(V_{\left(k+1\right)}^{T}K\left(X,X\right)V_{\left(k+1\right)}+\lambda I+\eta W_{\left(k\right)}^{T}W_{\left(k\right)}\right)}^{-1}\left(V_{\left(k+1\right)}^{T}K\left(X,X\right)+\eta W_{\left(k\right)}^{T}l\right)\;$$

Step 5: Iteration from Step 2 to Step 4 until convergence.

**Figure 6:**
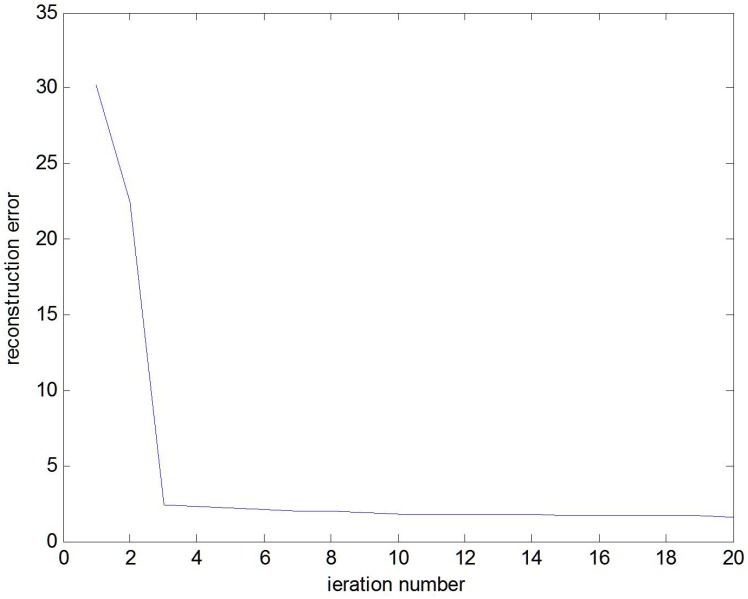
The representative reconstruction error of the objective function.

A whole algorithm summary, which includes the above optimization procedures, is given in Algorithm 1, and the representative reconstruction error of the objective function is shown in Figure 6. In case of the optimal A, we can derive the optimal solution of z based on Equation (7) as:

(15) $$z_{i}={\left(V^{T}K\left(X,X\right)V+\lambda I\right)}^{-1}V^{T}K\left(s_{i},X\right)\;$$

where $K\left(s_{i},X\right)=\left[\kappa \left(s_{i},x_{i}\right),\cdots ,\kappa \left(s_{i},x_{N}\right)\right]$.

**Algorithm 1. The Iteration Optimization Procedure.**Input:
$K\left(Y,Y\right)\in R^{N\times N}$
Output:$V\in R^{N\times K}$, $A\in R^{K\times N}$, $W\in R^{L\times m}$1.
**Initialization**: Randomly set $V_{\left(0\right)}$ with appropriate dimensions and obtain initial A according to Equation (8).2.
while Not convergent
do
3. Fixing
$A_{\left(k\right)}$, update $V_{\left(k+1\right)}$ according to Equation (10)4. Fixing
$V_{\left(k+1\right)}$ and $A_{\left(k\right)}$, update $W_{\left(k+1\right)}$ according to Equation (12)5. Fixing
$V_{\left(k+1\right)}$ and $W_{\left(k+1\right)}$, update $A_{\left(k+1\right)}$ according to Equation (14)6.
endwhile

## 4. Experiments

### 4.1. Dataset and Experiment Setup

**Figure 7:**
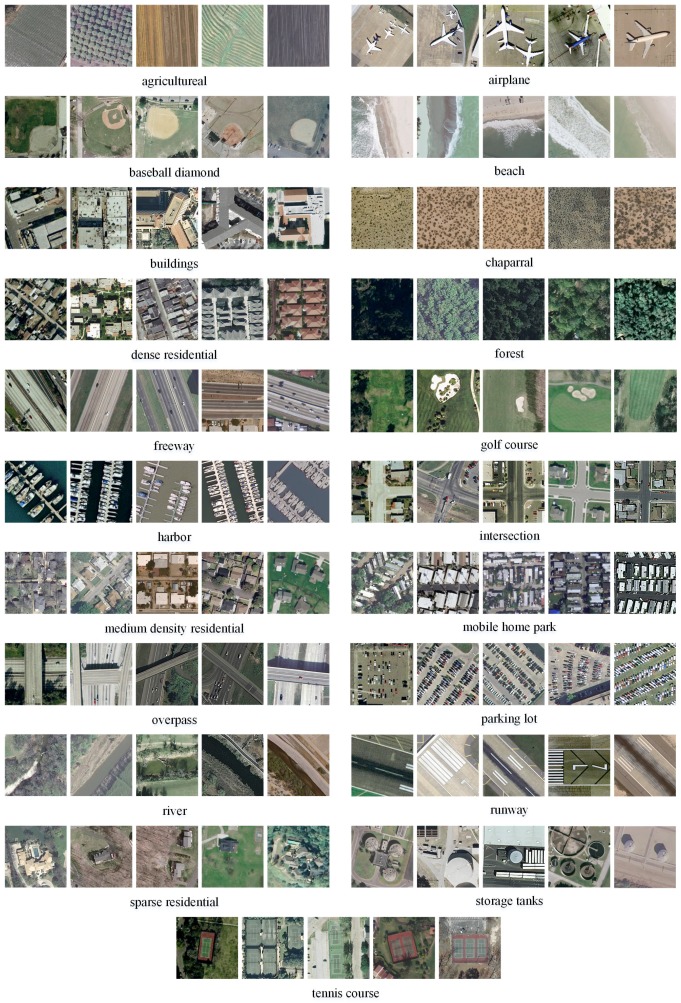
Samples from UCMERCED. Example geographic images associated with 21 categories are shown here.

In this section we demonstrate the application of our method in the classification experiments using a publicly available dataset [1], which includes twenty one scene categories with one hundred images of each class. This dataset corresponds to various land LULC types, which is shown in Figure 7.

For each category, it is randomly partitioned into five subsets, and each subset contains twenty samples. During the experiments, one subset is used for testing, and the remaining four subsets are used for training. Finally, we report the average classification accuracy.

### 4.2. Parameter Analysis

Equation (6) has four parameters, *λ*, *η*, *ρ* and dictionary size *K*, which need to be tuned. In order to determine their values, *n*-fold cross-validation is adopted. Each parameter is investigated by fixing the other parameters. It is noted that the initialization of *K* is 210.

**Figure 8:**
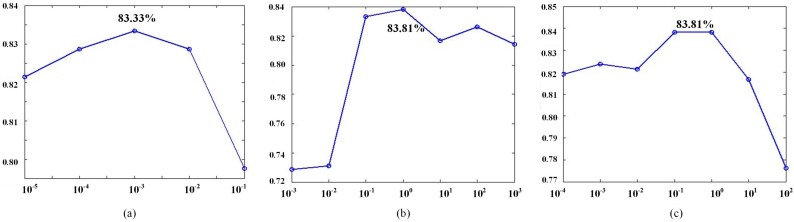
Evaluation of the effect on the classification accuracy for parameters: (**a**) *λ*; (**b**) *η*; and (**c**) *ρ*.

Figure 8 shows the classification accuracy of each tuned parameter. It is easy to find that our approach obtains the best performance (83.81%) when λ=0.001, η=1, ρ=0.1 (or 1).

### 4.3. Experiment Results and Comparison

The following three baseline methods are designed for comparison:

1. This method isolates the feature representation and classification process, which means that $A_{0}$ is used as the feature representation and that $W_{0}$ is used as the linear classifier.
2. This method is the same as the proposed method, except that the covd is established based on image intensities and the magnitude of the first and second gradients. Namely, $f_{x,y}={\left[c_{R,x,y}^{T},c_{G,x,y}^{T},c_{B,x,y}^{T}\right]}^{T}$ and $c_{C,x,y}=\left[I_{C,x,y},\sqrt{{\left(\frac{\partial I_{C}}{\partial x}\right)}^{2}+{\left(\frac{\partial I_{C}}{\partial y}\right)}^{2}},\sqrt{{\left(\frac{\partial^{2}I_{C}}{\partial^{2}x}\right)}^{2}+{\left(\frac{\partial^{2}I_{C}}{\partial^{2}y}\right)}^{2}}\right]$.
3. This method is the same as baseline Method 1, except that the covd is a 9 × 9 matrix, which is the same as baseline Method 2.

**Figure 9:**
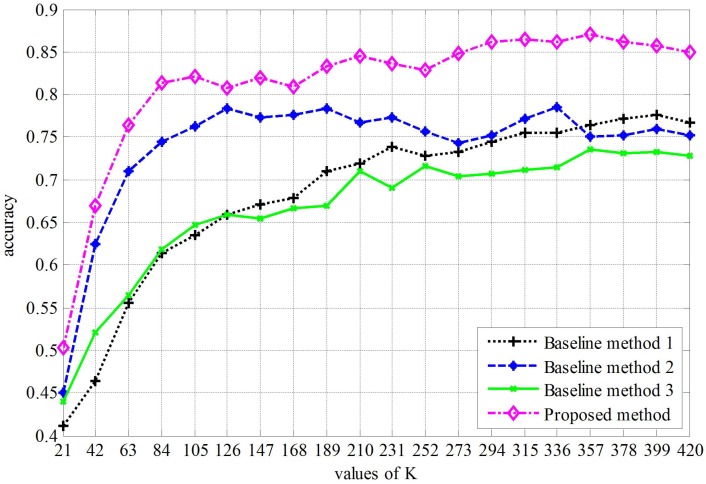
Comparison of different methods.

Figure 9 shows the classification accuracy *versus* dictionary size *K*. From this figure, we can find some interesting results:

1. Our approach is always better than the three baseline methods, and when K=357, our approach obtains the best performance (87.14%).
2. Comparing to baseline Method 1, our proposed method obtains a higher classification accuracy, which indicates the effectiveness of the optimization algorithm.
3. Comparing the proposed method to baseline Method 2, the only difference is the covd. The former uses a 15 × 15 matrix, which is a covariance format of intensity of each channel and the norms of the first and second gradients of intensities, while the covd of the latter is a 9 × 9 covariance format of the intensity of each channel and the magnitude of the first and second gradients. It is clear that both covds are not rotationally invariant, especially that the former covd is not direction invariant. However, the proposed method obtains a higher classification accuracy. This may indicate that the covariance format offsets the rotations to some extent.

**Figure 10:**
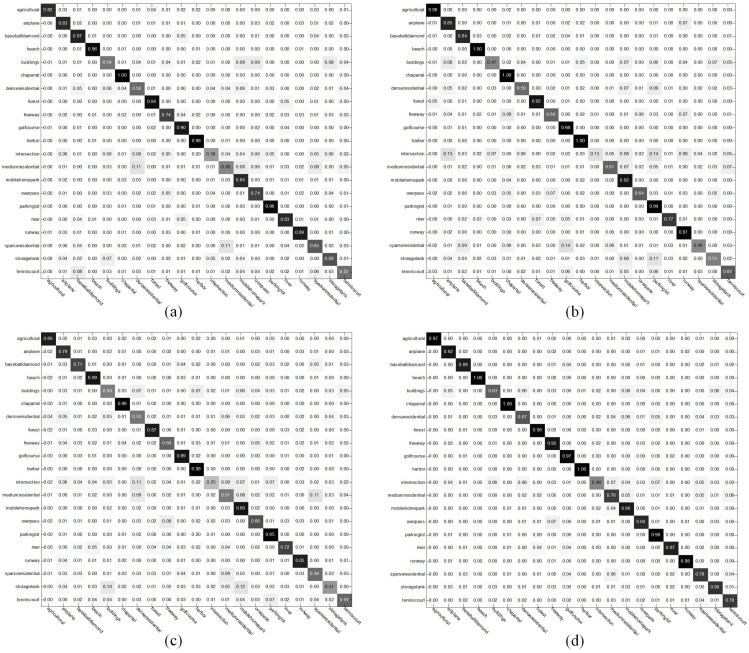
The average confusion matrices of: (**a**) baseline Method 1; (**b**) baseline Method 2; (**c**) baseline Method 3; and (**d**) the proposed method.

Figure 10 shows the confusion matrices of the baseline methods and our approach, respectively. The classification accuracy of fifteen categories is more than 80%, and eleven categories are more than 90%. Nevertheless, the classification accuracy of three categories, buildings, dense residential and intersection, is less than 70%.

**Figure 11:**
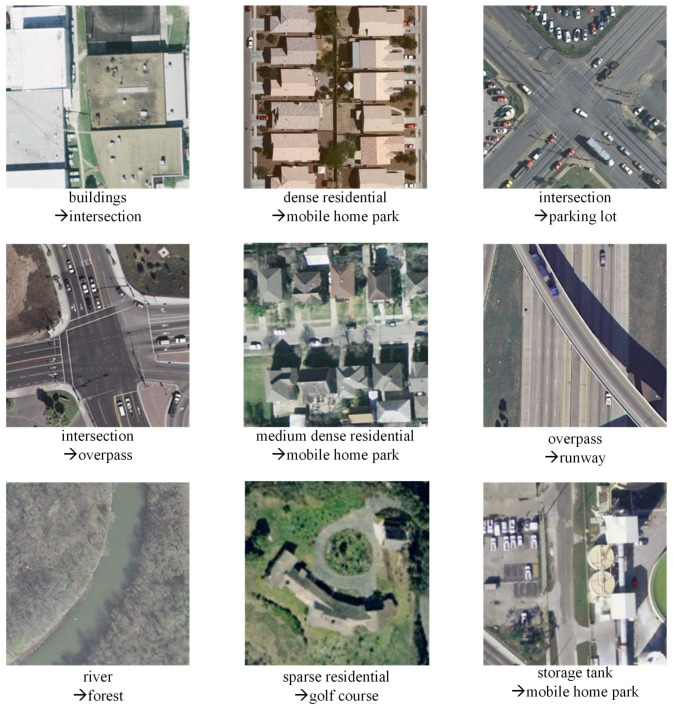
The representative misclassification samples.

In order to analyze the proposed method, Figure 11 lists some representative misclassification samples of the proposed method. Some misclassification couples, such as intersection/overpass, overpass/runway and river/forest, shown in Figure 11 are hard to identify, even with our own eyes.

**Figure 12:**
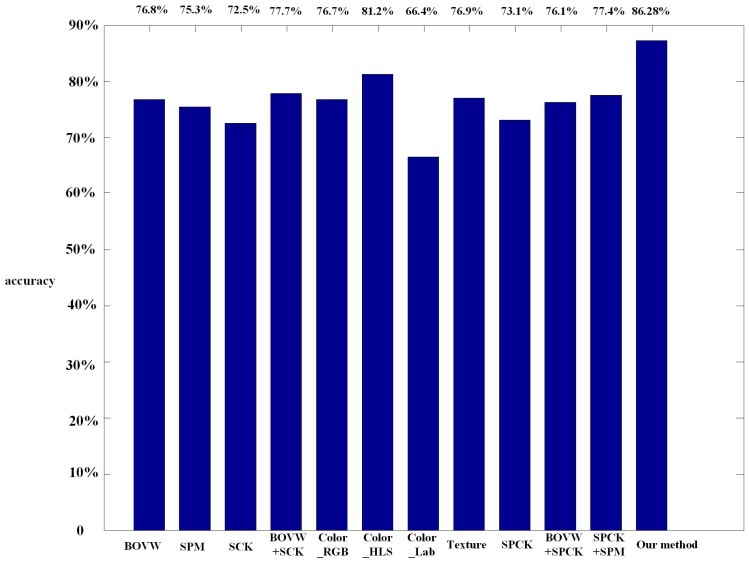
The comparison of our method with the state-of-the-art performance reported in the literature on the dataset UCMERCED. BOVW, bag of visual words; SPM, spatial pyramid matching; SCK, spatial co-occurrence kernel; SPCK, spatial pyramid co-occurrence kernel.

Besides, we report the classification accuracies of both baseline methods and our method over groups rotated five times in Table 1. Then, the comparison with the classical approaches [1,2], BOVW, SPM, SCK, BOVW + SCK, color histogram, such as RGB, HLS and CIE Lab, texture, SPCK, BOVW + SPCK and SPCK + SPM, is shown in Figure 12.

## 5. Conclusions

This paper proposes a novel supervised collaborative kernel coding model based on covd for scene-level geographic image classification. Since covd lies in non-Euclidean space, the linear classifier, which is based on Euclidean distance, cannot be utilized. Additionally, our main contribution is explicitly integrating the discriminative feature coding and a linear classifier into the objective function. Moreover, the solution to the new objective function is efficiently achieved by simply employing the optimization algorithm. Experiments implemented on the UCMERCED dataset show the effectiveness of our approach.

## Figures and Tables

**Table 1:** classification accuracies over all five groups of our method.

Subset Number	1	2	3	4	5	Average
baseline Method 1	78.10%	79.29%	76.90%	78.81%	71.90%	77.00%
baseline Method 2	78.33%	75.48%	74.29%	75.71%	74.29%	75.62%
baseline Method 3	73.10%	71.43%	70.00%	73.33%	69.05%	71.38%
proposed Method	87.14%	84.52%	88.10%	87.14%	84.52%	86.28%

## References

[1] Yang Y., Newsam S. Bag-of-visual-words and spatial extensions for land-use classification. Proceedings of the 18th SIGSPATIAL International Conference Advances in Geographic Information Systems.

[2] Yang Y., Newsam S. Spatial pyramid co-occurrence for image classification. Proceedings of the 7th IEEE International Conference on Computer Vision.

[3] Xu S., Fang T., Wang S. (2010). Object classification of aerial images with bag-of-visual words. IEEE Geosci. Remote Sens. Lett..

[4] Aksoy S., Koperski K., Tusk C., Marchisio G., Tilton J.C. (2005). Learning bayesian classifiers for scene classification with a visual grammar. IEEE Trans. Geosci. Remote Sens..

[5] Yang Y., Newsam S. (2013). Geographic image retrieval using local invariant features. IEEE Trans. Geosci. Remote Sens..

[6] Schroder M., Rehrauer H., Seidel K., Datcu M. (2000). Interactive learning and probabilistic retrieval in remote sensing image archives. IEEE Trans. Geosci. Remote Sens..

[7] Shyu C., Klaric M., Scott G.J., Barb A.S., Davis C.H., Palaniappan K. (2000). GeoIRIS: Geospatial information retrieval and indexing system-content mining, semantics modeling and complex queries. IEEE Trans. Geosci. Remote Sens..

[8] Kim M., Madden M., Warner T.A. (2000). Forest type mapping using object-specific texture measures from multispectral Ikonos imagery: Segmentation quality and image classification issues. Photogramm. Eng. Remote Sens..

[9] Zhang Y., Wu L., Neggaz N., Wang S., Wei G. (2009). Remote-sensing image classification based on an improved probabilistic neural network. Sensors.

[10] Cheriyadat A. (2014). Unsupervised feature learning for aerial scene classification. IEEE Trans. Geosci. Remote Sens..

[11] Du P., Xia J., Zhang W., Tan K., Liu Y., Liu S. (2012). Multiple classifier system for remote sensing image classification: A review. Sensors.

[12] Cheng G., Han J., Zhou P., Guo L. (2014). Multi-class geospatial object detection and geographic image classification based on collection of part detectors. ISPRC J. Photogramm. Remote Sens..

[13] Li J., Du Q., Li W., Li Y. (2015). Optimizing extreme learning machine for hyperspectral image classification. J. Appl. Remote Sens..

[14] Csurka G., Dance C., Fan L., Willamowski J., Bray C. (2004). Visual categorization with bags of keypoints. Proceedings of the ECCV International Workshop on Statistical Learning in Computer Vision.

[15] Cao Y., Wang C., Li Z., Zhang L.Q., Zhang L. Spatial-bag-of-features. Proceedings of the IEEE Conference on Computer Vision and Pattern Recognition.

[16] Lazebnik S., Schmid C., Ponce J. Beyond bags of features: Spatial pyramid matching for recognizing natural scene categories. Proceedings of the IEEE Computer Society Conference on Computer Vision and Pattern Recognition.

[17] Yang J., Yu K., Gong Y., Huang T. Linear spatial pyramid matching suing sparse coding for image classification. Proceedings of the IEEE Computer Society Conference on Computer Vision and Pattern Recognition.

[18] Tuzel O., Porikli F., Meer P. Region covariance: A fast descriptor for detection and classification. Proceedings of the European Conference on Computer Vision.

[19] Erdem E., Erdem A. (2013). Visual saliency estimation by nonlinearly integrating features using region covariances. J. Vis..

[20] Tuzel O., Porikli F., Meer P. (2008). Pedestrian detection via classification on riemannian manifolds. IEEE Trans. Pattern Anal. Mach. Intell..

[21] Porikli F., Tuzel O., Meer P. Covariance tracking using model update based on lie algebra. Proceedings of the IEEE Computer Society Conference on Computer Vision and Pattern Recognition.

[22] Wang R., Guo H., Davis L., Dai Q. Covariance discriminative learning: A natural and efficient approach to image set classification. Proceedings of the IEEE Conference on Computer Vision and Pattern Recognition.

[23] Wang L., Liu H., Sun F. (2016). Dynamic texture video classification using extreme learning machine. Neurocomputing.

[24] Arsigny V., Fillard P., Pennec X., Ayache N. (2006). Geometric means in a novel vector space structure on symmetric positive-definite matrices. SIAM J. Matrix Anal. Appl..

[25] Arsigny V., Fillard P., Pennec X., Ayache N. (2006). Log-euclidean metrics for fast and simple calculus on diffusion tensors. Magn. Reson. Med..

[26] Li P., Wang Q., Zhang L. Log-euclidean kernels for sparse representation and dictionary learning. Proceedings of the IEEE International Conference on Computer Vision.

[27] Bo L., Sminchisescu C. Efficient match kernels between sets of features for visual recognition. Proceedings of the Advances in Neural Information Processing Systems.

[28] Gao S., Tsing I., Chia L. (2013). Sparse representation with kernels. IEEE Trans. Image Process..

[29] Harandi M., Salzmann M. Riemannian coding and dictionary learning: Kernels to the rescue. Proceedings of the IEEE Computer Society Conference on Computer Vision and Pattern Recognition.

[30] Van Nguyen H., Patel V.M., Nasrabadi N.M., Chellappa R. (2013). Design of non-linear kernel dictionaries for object recognition. IEEE Trans. Image Process..

[31] Kim M. (2014). Efficient kernel sparse coding via first-order smooth optimization. IEEE Trans. Neural Netw. Learn. Syst..

